# The SnRK2 kinases modulate miRNA accumulation in *Arabidopsis*

**DOI:** 10.1371/journal.pgen.1006753

**Published:** 2017-04-18

**Authors:** Jun Yan, Pengcheng Wang, Bangshing Wang, Chuan-Chih Hsu, Kai Tang, Hairong Zhang, Yueh-Ju Hou, Yang Zhao, Qiming Wang, Chunzhao Zhao, Xiaohong Zhu, W. Andy Tao, Jianming Li, Jian-Kang Zhu

**Affiliations:** 1 Shanghai Center for Plant Stress Biology, and Center for Excellence in Molecular Plant Sciences, Chinese Academy of Sciences, Shanghai, China; 2 Department of Horticulture and Landscape Architecture, Purdue University, West Lafayette, Indiana, United States of America; 3 Departments of Biochemistry, Purdue University, West Lafayette, Indiana, United States of America; 4 State Key Laboratory of Wheat and Maize Crop Sciences, College of Life Science, Henan Agricultural University, Zhengzhou, China; 5 College of Biosciences and Biotechnology, Hunan Agricultural University, Changsha, China; Swedish University of Agricultural Sciences (SLU), SWEDEN

## Abstract

MicroRNAs (miRNAs) regulate gene expression and play critical roles in growth and development as well as stress responses in eukaryotes. miRNA biogenesis in plants requires a processing complex that consists of the core components DICER-LIKE 1 (DCL1), SERRATE (SE) and HYPONASTIC LEAVES (HYL1). Here we show that inactivation of functionally redundant members of the SnRK2 kinases, which are the core components of abscisic acid (ABA) and osmotic stress signaling pathways, leads to reduction in miRNA accumulation under stress conditions. Further analysis revealed that the steady state level of HYL1 protein in plants under osmotic stress is dependent on the SnRK2 kinases. Additionally, our results suggest that the SnRK2 kinases physically associate with the miRNA processing components SE and HYL1 and can phosphorylate these proteins in vitro. These findings reveal an important role for the SnRK2 kinases in the regulation of miRNA accumulation and establish a mechanism by which ABA and osmotic stress signaling is linked to miRNA biogenesis.

## Author summary

miRNAs play critical roles in various biological processes. miRNA biogenesis in both animals and plants are generated by a miRNA processing complex. Studies on specific miRNA function in different biological process have been extensively reported. However, how miRNA biogenesis is modulated, especially by environmental stimuli is poorly understood. The family of 10 redundant members of SnRK2 protein kinases are the central components of abscisic acid (ABA) and osmotic stress signaling pathways. In this study, our work reveals a novel role for SnRK2s in the modulation of miRNA levels. Importantly, we show that the SnRK2 kinases are required for the maintenance of the steady state level of HYL1 protein and the miRNA processing complex components, SE and HYL1, are potential substrates of the SnRK2 kinases. Our study provides a new mechanism of regulation of miRNA biogenesis and paves the way for further understanding of the impact of ABA and osmotic stress signaling on miRNA production.

## Introduction

MicroRNAs (miRNAs) are non-coding RNAs that impact various biological processes by regulating mRNA cleavage or translation in a sequence-specific manner. Mature miRNAs are generated by the miRNA processing complex. In plants, the core components of miRNA processing complex include the RNAse III enzyme DICER-LIKE 1 (DCL1), the zinc finger protein SERRATE (SE) and the double-stranded RNA binding protein HYPONASTIC LEAVES (HYL1) [1–10]. DAWDLE (DDL), TOUGH (TGH), Negative on TATA less 2 (NOT2), CELL DIVISION CYCLE 5 (CDC5), PRL1 and CDF2, which are associated with DCL1, also function in miRNA biogenesis [11–16]. Several proteins, CBP80/ABH1, HOS5/SHI1/RCF3, RS40 and RS41, C-TERMINAL DOMAIN PHOSPHATASE-LIKE 1 (CPL1)/FIERY2 (FRY2), RECEPTOR FOR ACTIVATED C KINASE 1 (RACK1), which interact with SE or HYL1, are also required for miRNA generation [17–21]. STABLIZED 1 (STA1) and XAP5 CIRCADIAN TIMEKEEPER (XCT) can modulate miRNA accumulation through regulation of *DCL1* transcript level [22, 23]. Several other proteins, such as SICKLE (SIC), Modifier of SNC1, 2 (MOS2), AtGRP7 and THO2 are additional players involved in miRNA accumulation [24–27]. miRNAs in both animals and plants are processed from primary miRNA (pri-miRNA) transcripts, which include imperfect stem-loop structures. In animals, pri-miRNAs are cleaved in the nucleus by the endonuclease Drosha to generate precursor miRNAs (pre-miRNAs), which are then further processed into mature miRNAs by DICER in the cytoplasm [2]. In plants, the processing of both pri-miRNAs and pre-miRNAs is catalyzed by DCL1 in the nucleus [1, 3]. Previous studies demonstrated important roles of protein phosphorylation in regulating miRNA biogenesis in Arabidopsis [11, 20, 28]. Maintenance of the hypophosphorylated state of HYL1 by CPL1 is required for accurate and efficient miRNA processing [20]. However, little is known about the kinases that may phosphorylate and regulate the core components of the *Arabidopsis* miRNA processing complex. A recent study suggested that HYL1 is a putative substrate of the mitogen-activated protein kinase MPK3 in both *Arabidopsis* and rice [29].

In eukaryotes, protein kinases are key regulators of almost all cellular processes and play prominent roles in various signal transduction pathways. The yeast Sucrose non-fermenting (Snf1) protein kinase, the mammalian AMP-activated protein kinase and the plant SNF1-related protein kinase (SnRKs) are highly conserved and are the central components of kinase cascades [30]. In *Arabidopsis*, SnRK subfamily 2 (SnRK2s) is a 10-member family (SnRK2.1–2.10) [31]. SnRK2.2, SnRK2.3 and SnRK2.6 belong to the subgroup III SnRK2 family and are strongly activated by ABA, while all SnRK2s except SnRK2.9 can be activated by osmotic stress [32]. SnRK2.2, 2.3 and 2.6 act redundantly in ABA regulation of stomatal closure, seed germination and seedling growth, and virtually all examined ABA responses are eliminated in *snrk2*.*2/3/6* triple mutants [33, 34]. Interestingly, the *snrk2* decuple mutant is similar to the wild-type control on culture medium with high humidity, but displays severe growth defects in soil and in culture media under osmotic stress and shows great reduction in osmotic-stress-induced responses [35].

The functional specificity of SnRK2s in different biological processes is conferred through SnRK2s-mediated phosphorylation of their diverse substrates. The best-characterized substrates of SnRK2s are ABA-responsive element binding proteins (AREBs) [36, 37]. SnRK2-catalyzed phosphorylation of AREBs is critical for activating the transcription of ABA responsive genes. Similarly, ABA-induced stomatal closure requires SnRK2-dependent phosphorylation of SLAC1 (Slow Anion Channel-Associated 1) that is critical for ion efflux from guard cells [38, 39]. SNS1 (SnRK2-substrate 1), a recently identified SnRK2s substrate, functions as a negative regulator of ABA signaling during post-germination growth [40]. Other characterized SnRK2 substrates includes the NADPH oxidase RbohF essential for generating reactive oxygen species (ROS) in response to ABA, the potassium channel KAT1 (K+ channel in *Arabidopsis thaliana* 1) involved in ABA regulation of stomatal movement, the aquaporin PIP2;1 (Plasma membrane Intrinsic Protein 2;1) and the SWI/SNF chromatin-remodeling ATPase BRAHMA (BRM) in the modulation of ABA responses [41–44].

In this study, we report that SnRK2s are required for proper miRNA accumulation by regulation of miRNA processing factors. Our study provides new insights into the molecular mechanism underlying the regulation of miRNA biogenesis and furthers our understanding of the impact of ABA and osmotic stress signaling on plant growth and development through regulation of miRNA accumulation.

## Results

### The SnRK2 kinases are required for the accumulation of miRNAs

**Fig 1:**
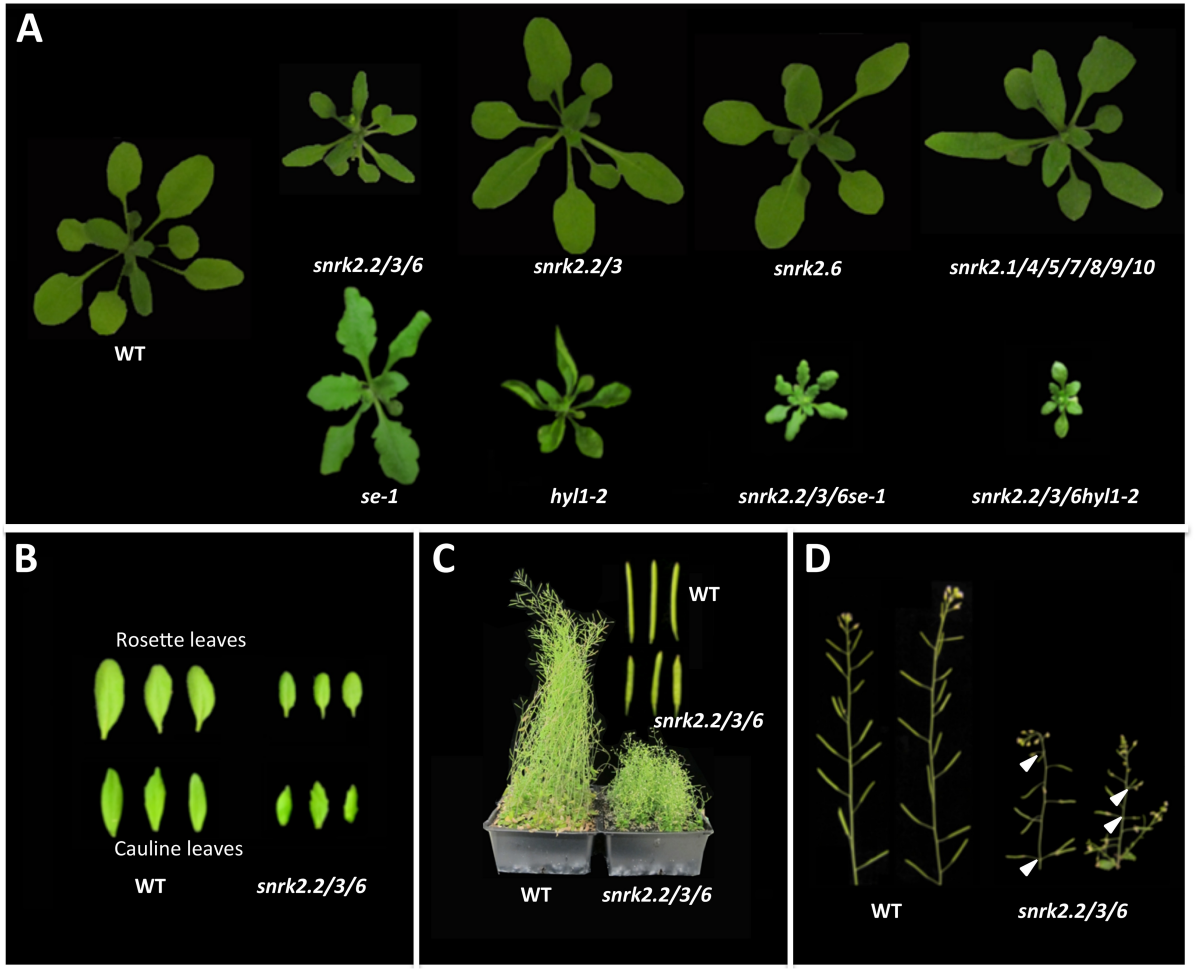
Soil-grown *snrk2*.*2/3/6* mutant exhibits some miRNA deficiency morphological defects. (A) Comparison of the phenotype of 3-week-old plants of different genotypes grown in soil under normal growth room conditions. (B) Comparison of wild type and *snrk2*.*2/3/6* mutant rosette and cauline leaves. (C) Comparison of the plant stature and siliques of 2-month-old wild type and *snrk2*.*2/3/6* mutant. (D) The *snrk2*.*2/3/6* mutant exhibits abnormal silique phyllotaxy. Arrow heads indicate altered silique phyllotaxy of *snrk2*.*2/3/6* mutant.

**Fig 2:**
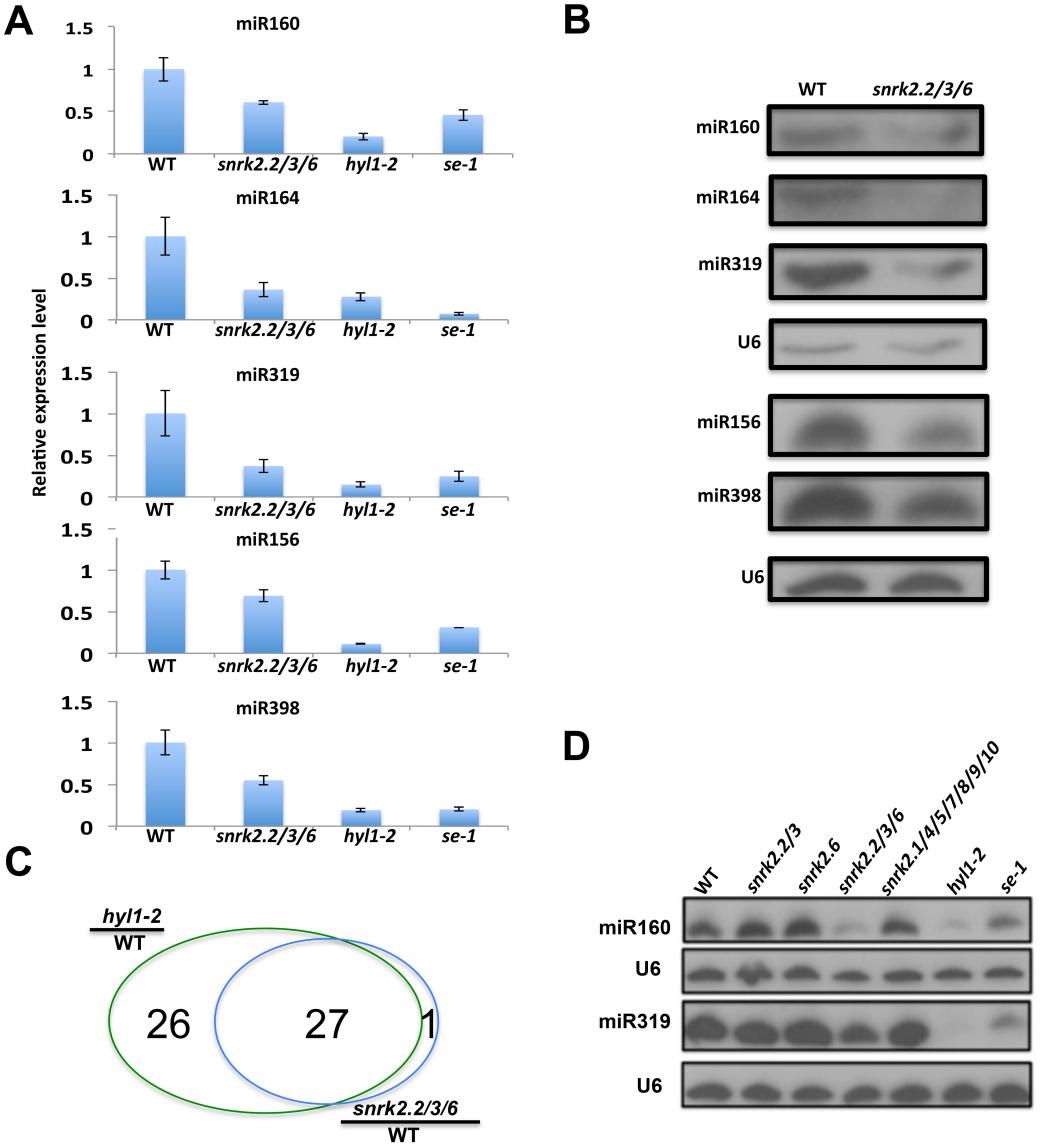
Reduction of miRNA abundance in soil-grown *snrk2*.*2/3/6* mutant. (A) qRT-PCR analysis of the expression of mature miRNAs in rosette leaves of wild type, *snrk2*.*2/3/6*, *hyl1-2* and *se-1*. qRT-PCR results are means ±SD of three biological replicates where the fold changes are normalized to the transcript level in WT. (B) RNA blot hybridization of miRNAs from rosette leaves of 3-week-old wild type, *snrk2*.*2/3/6*, *hyl1-2* and *se-1*. U6 small nuclear RNA was used as loading control. (C) Comparison of identical miRNAs with >2-fold abundance reduction (relative to the wild-type control) between *hyl1-2* and *snrk2*.*2/3/*6 mutants. (D) Expression of selected miRNAs in rosette leaves of 3-week-old wild type, *snrk2*.*2/2*.*3*, *snrk2*.*6*, *snrk2*.*2/3/6*, *snrk2*.*1/4/5/7/8/9/10*, *hyl1-2* and *se-1*.

During the characterization of the *snrk2*.*2/3/6* triple mutant, we noticed that compared with its wild type control, both the rosette and cauline leaves are smaller, and older rosette leaves and cauline leaves are serrated in the *snrk2*.*2/3/6* mutant under our plant growth room conditions (Fig 1A and 1B and S1 Fig). Additionally, the *snrk2*.*2/3/6* mutant is a dwarf with its siliques being shorter and arranged abnormally (Fig 1C and 1D). Some of these morphological variations shared features with those of miRNA deficient mutants such as *se-1* and *hyl1-2*. When the *se-1* or *hyl1-2* mutation was introduced into the *snrk2* triple mutant, the resulting *snrk2*.*2/3/6se-1* and *snrk2*.*2/3/6hyl1-2* quadruple mutants were similar to *snrk2*.*2/3/6* in their small size but resembled *se-1* and *hyl1-2*, respectively, in their leaf morphology (Fig 1A). The shared morphological defects between *snrk2*.*2/3/6* and miRNA deficient mutants prompted us to test whether accumulation of miRNAs is affected in *snrk2*.*2/3/6* mutant plants. We randomly chose several miRNAs to evaluate their levels in rosette leaves using quantitative real-time reverse-transcription PCR (qRT-PCR) and found that the amounts of miR160, miR164, miR319, miR156 and miR398 were reduced in *snrk2*.*2/3/6* mutant compared with those of the wild type control (Fig 2A). Small RNA blot analysis was subsequently conducted to further examine the accumulation of these miRNAs. Consistent with the qRT-PCR results, the abundance of all these miRNAs was reduced in the *snrk2*.*2/3/6* mutant (Fig 2B), indicating that the lack of the SnRK2s affects the abundance of the tested miRNAs. We also compared the abundance of 24-nt siRNAs in the *snrk2*.*2/3/6* mutant and its wild type control but found no substantial difference (S2 Fig).

To further investigate whether the SnRK2s has effects on additional miRNAs, miRNA expression profile of *snrk2*.*2/3/6* was compared with those of wild type and *hyl1-2* plants using small RNA deep sequencing. Only highly expressed miRNAs with at least 10 reads in normalized counts in wild type were selected for analysis. We found that 53 miRNAs in *hyl1-2* mutant, and 28 miRNAs in *snrk2*.*2/3/6* show at least a two-fold reduction compared to the wild type (S3 Fig). Interestingly, 27 miRNAs with a pronounced reduction in *snrk2*.*2/3/6* mutant were reduced in *hyl1-2* mutant (Fig 2C and S3 Fig). We analyzed misprocessed miRNAs in wild type, *hy1-2* and *snrk2*.*2/3/6* mutants. We focused on the highly expressed miRNAs (miRNAs with at least 10 reads in each replicate) given that the evaluation of miRNA precision depends on sequencing depth. We found that miRNA processing accuracy is affected in the *hyl1-2* mutant as expected, but the processing accuracy is not substantially affected in the *snrk2*.*2/3/6* mutant (S4 Fig). These results indicate that the SnRK2s are important for miRNA biogenesis.

### Members of the SnRK2 kinase subfamily III act redundantly in regulating miRNA biogenesis

At the vegetative stage, although *snrk2*.*2/3/6* mutant showed severe morphological defects, mutations in the other seven members of SnRK2s did not cause obvious morphological defects. Additionally, no obvious morphological difference was observed among *snrk2*.*6* single mutant, *snrk2*.*2/2*.*3* double mutant, and the wild type (Fig 1A). To determine the contributions of the SnRK2 family members in miRNA biogenesis, the levels of two miRNAs, miR160 and miR319, were further compared in the rosette leaves of soil grown wild type, *snrk2*.*2/2*.*3*, *snrk2*.*6*, and *snrk2*.*1/4/5/6/7/8/9/10* mutants. Although the expression levels of these miRNAs were substantially reduced in the *snrk2*.*2/3/6* triple mutant (Fig 2D), no substantial difference was found in the single (*snrk2*.*6*), double (*snrk2*.*2/3*) or septuple mutants compared with the wild type control (Fig 2D). These results strongly suggest functional redundancy among the three members of the SnRK2 subgroup III in the regulation of miRNA biogenesis under the tested conditions.

### The SnRK2 kinases are involved in the processing of pri-miRNAs

**Fig 3:**
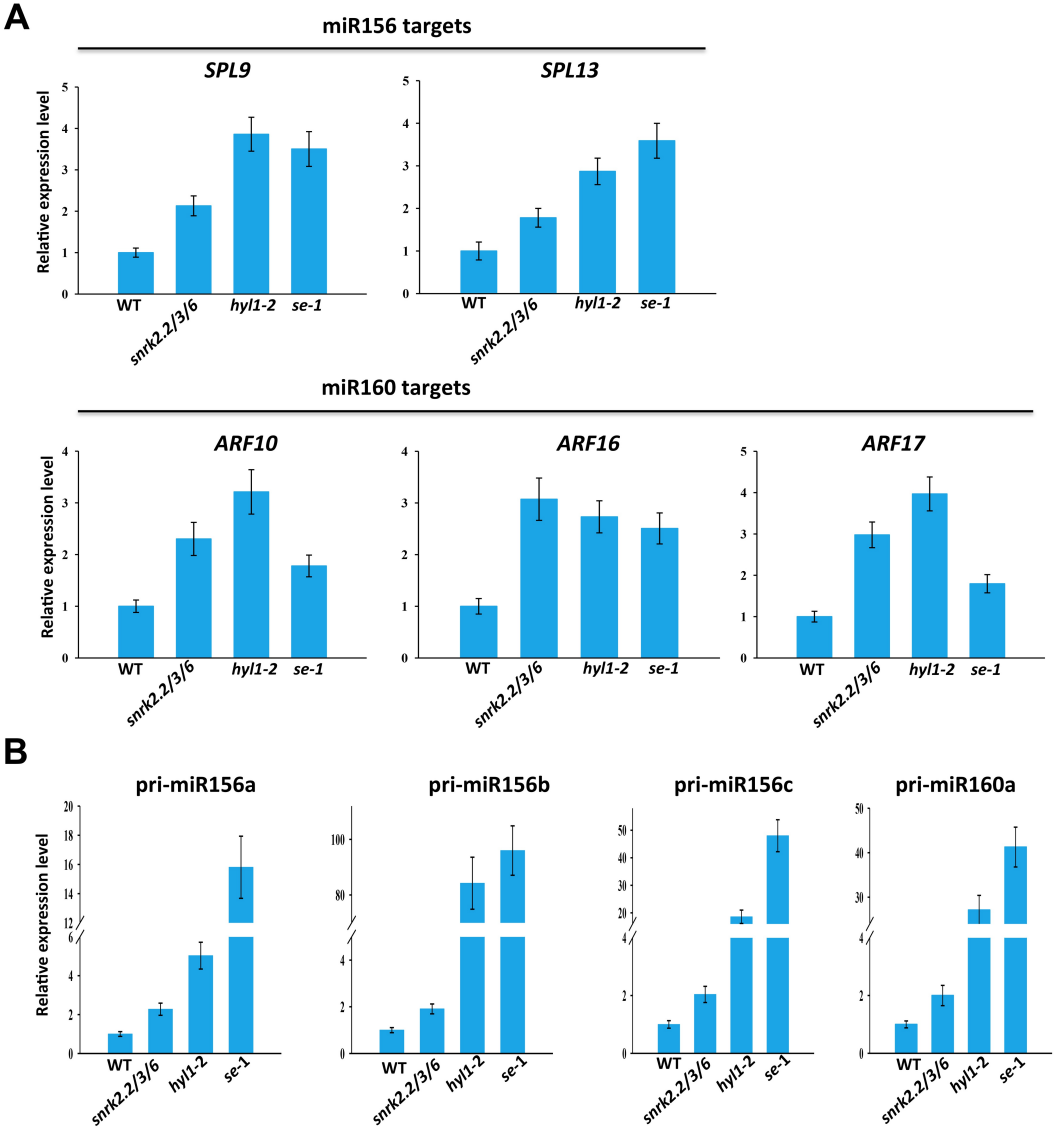
Increased accumulation of miRNA targets and pri-miRNAs in soil-grown *snrk2*.*2/3/6* mutant. (A) qRT-PCR analysis of the expression of miR156 and miR160 targets in rosette leaves of wild type and *snrk2*.*2/3/6*. (B) qRT-PCR analysis of the expression of pri-miRNAs in rosette leaves of wild type and *snrk2*.*2/3/6*. In (A) and (B), qRT-PCR results are means ±SD of three biological replicates where the fold changes are normalized to the transcripts level in WT.

Since miRNAs can negatively regulate the accumulation of target mRNAs, we measured the amount of target mRNAs for some of the miRNAs in soil grown wild type and *snrk2*.*2/3/6* mutant using qRT-PCR. As expected, increased target transcript levels were observed in the *snrk2*.*2/3/6* mutant (Fig 3A). To investigate whether the SnRK2 kinases are involved in pri-miRNA processing, the pri-miRNA transcript levels were determined by qRT-PCR. We found that the accumulation of the tested pri-miRNAs accumulated to higher levels in *snrk2*.*2/3/6* mutant than wild type (Fig 3B), indicating that the SnRK2 kinases are required for the proper processing of pri-miRNAs.

### Inactivation of the SnRK2 kinases affects miRNA biogenesis under ABA treatment

**Fig 4:**
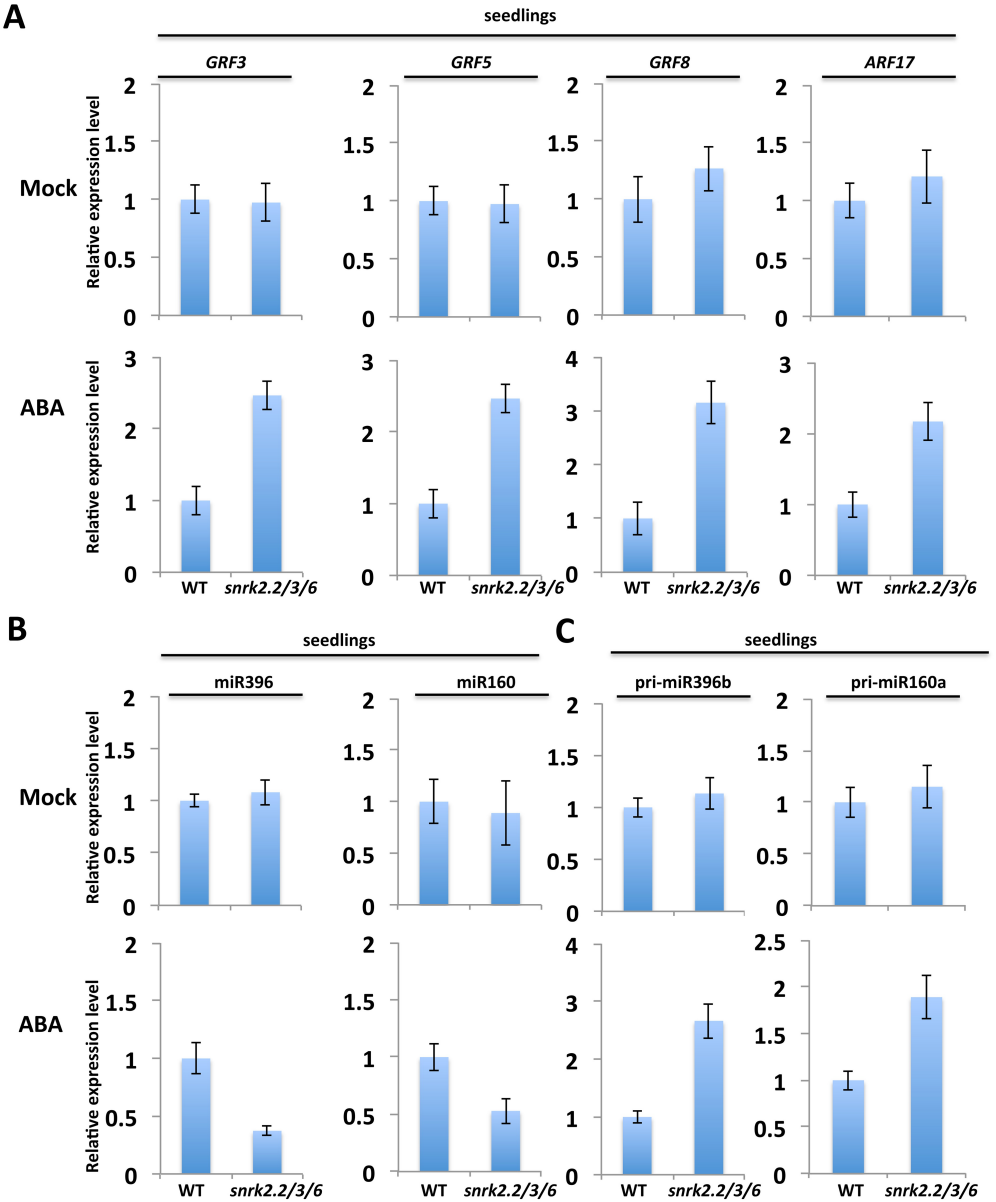
Effects of the triple *snrk2*.*2/3/6* mutations on the expression of miRNAs and their targets and precursors under ABA treatment. (A) qRT-PCR analysis of the expression of three miR396 targets, *GRF3*, *GRF5*, *GRF8* and *ARF17* in 2-week-old seedlings treated with or without 50 μM ABA. (B) qRT-PCR analysis of the expression of miR396 and miR160 in 2-week-old seedlings treated with or without 50 μM ABA. (C) qRT-PCR analysis of the transcript abundance of the pri-miR396b and pri-miR160a in 2-week-old seedlings treated with or without 50 μM ABA. In (A)-(C), qRT-PCR results are means ±SD of three biological replicates where the fold changes are normalized to the transcripts level in WT.

Because the three subgroup III SnRK2s were known to be activated by ABA, we also examined the effects of the *snrk2*.*2/3/6* triple mutations on miRNA expression under ABA treatment in seedlings grown in culture medium. We first analyzed publicly available microarray data on ABA-treated seedlings of *snrk2*.*2/3/6* and wild-type [45]. We compared the expression levels of experimentally validated miRNA targets, and found that many known miRNA target genes were upregulated, albeit to different extents, in ABA-treated *snrk2*.*2/3/6* mutant (S5 Fig). Many of those miRNA target genes belong to the same gene family, suggesting that their upregulation in the triple *snrk2*.*2/3/6* mutant might be attributed to the release of miRNA mediated repression. To test this possibility, we performed qRT-PCR analysis of the transcript abundance of *Growth-Regulating Factor 3* (*GRF3*), *GRF5* and *GRF8*, the well-defined target genes of miR396, and the miR160 target *Auxin Response Factor 17* (*ARF17*) in wild type and *snrk2*.*2/3/6* mutant. While no significant difference in their transcript levels was detected between the triple mutant and its wild type control without ABA treatment, their transcript levels were significantly higher in ABA-treated triple mutant seedlings compared to ABA-treated wild type in the culture medium (Fig 4A). Consistently, our qRT-PCR analysis revealed no obvious difference in miR396 and miR160 abundance between mock-treated wild-type and *snrk2*.*2/3/6* seedlings but a significant lower abundance of miR396 and miR160 in ABA-treated triple mutant compared to ABA-treated wild-type in the culture medium (Fig 4B). This analysis indicates that the increased transcript levels of *GRF*s and *ARF17* in *snrk2*.*2/3/6* mutant under ABA treatment are at least partially attributed to the reduced levels of miR396 and miR160, respectively. We also analyzed the abundance of the miRNA precursors, and found that pri-miR396b and pri-miR160a accumulated to higher levels in ABA-treated *snrk2*.*2/3/6* mutant than the ABA-treated wild type in the culture medium (Fig 4C). Taken together, these results support that the ABA-activated SnRK2 kinases regulate miRNA biogenesis.

### The SnRK2 kinases-mediated pathway modulates the level of the miRNA processing factor HYL1

**Fig 5:**
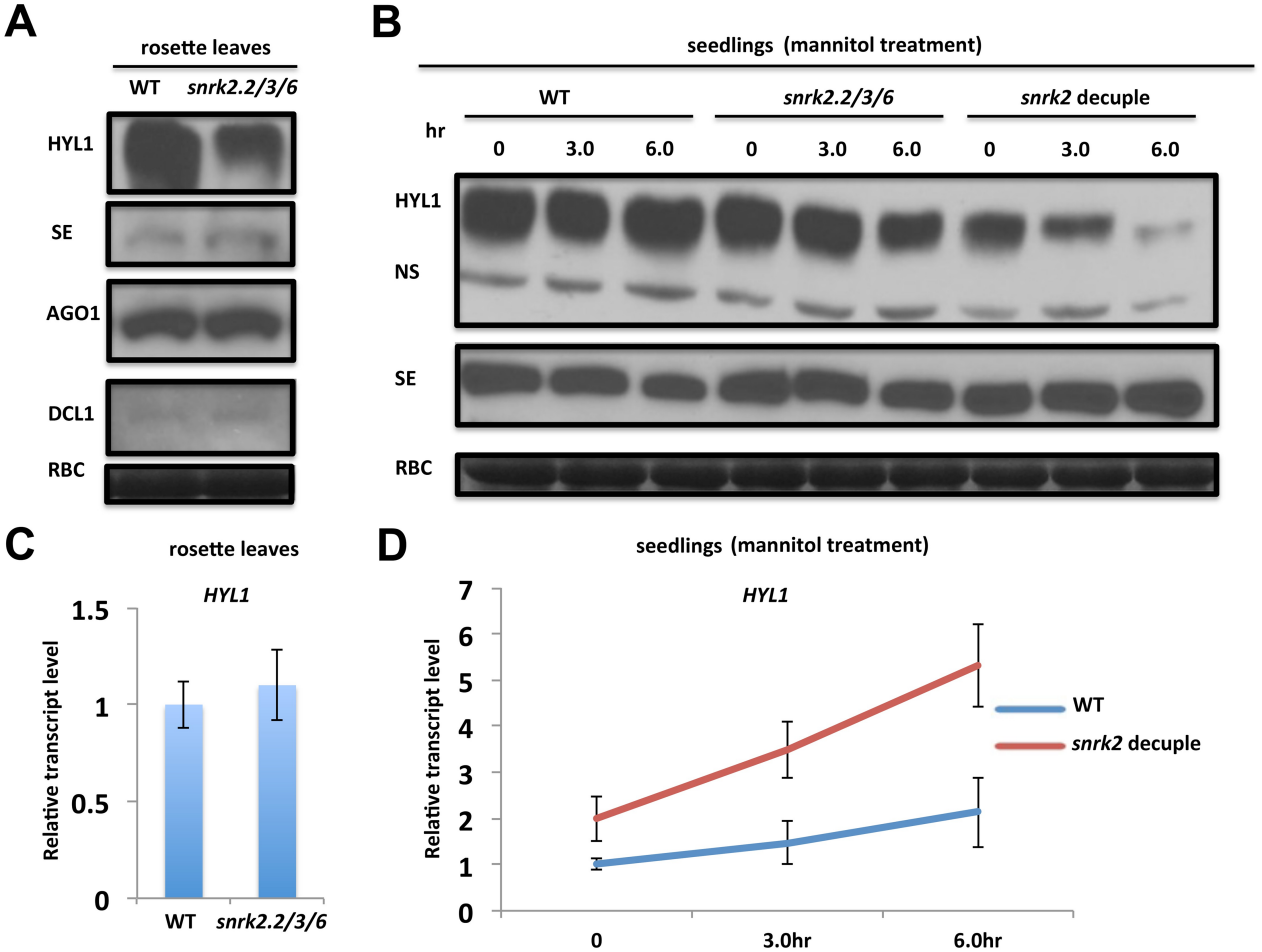
The effect of *snrk2* mutation on the expression of HYL1. (A) Immunoblot analysis of the protein abundance of key components of the miRNA biogenesis pathway in soil-grown wild type and *snrk2*.*2/3/6* mutant rosette leaves. (B) Immunoblot analysis of the protein abundance of HYL1 and SE in wild type and decuple mutant seedlings treated with or without 800 mM mannitol. In both (A) and (B), the Coomassie blue staining of the large subunit of Rubisco (RBC) serves as the loading control. NS in (B) indicates a non-specific band. (C) qRT-PCR analysis of the *HYL1* transcript level in wild type and *snrk2*.*2/3/6* mutant rosette leaves. (D) qRT-PCR analysis of the relative *HYL1* transcript abundance in wild type and *snrk2* decuple mutant seedlings treated with 800 mM mannitol for different durations.

To investigate potential mechanisms by which SnRK2s affect miRNA biogenesis, we first examined the protein abundance of key components of the *Arabidopsis* miRNA biogenesis pathway in the soil-grown *snrk2* triple mutant and its wild type control. Interestingly, while no substantial difference in the protein abundance of DCL1, AGO1 and SE was detected in rosette leaves between the two genotypes grown in soil, the HYL1 protein level was reduced substantially in the *snrk2* triple mutant (Fig 5A). Surprisingly, no significant difference in the HYL1 protein abundance was detected between whole seedlings of wild type and the triple mutant grown in culture medium (Fig 5B), suggesting involvement of additional SnRK2s in regulating the HYL1 abundance in cultured seedlings. Indeed, the HYL1 abundance was significantly reduced in the *snrk2* decuple mutant seedlings (Fig 5B). We also tested the impact of osmotic stress, which is known to activate nearly all 10 SnRK2s, on the HYL1 protein abundance of cultured seedlings. As shown in Fig 5B, although the 6-h mannitol treatment had little impact on the HYL1 abundance in wild type seedlings, a similar treatment slightly and dramatically reduced the HYL1 abundance in the cultured seedlings of the *snrk2* triple mutant and decuple mutant, respectively (Fig 5B), providing additional support for the involvement of other SnRK2s in regulating the HYL1 protein abundance. Consistent with this, we found that some miRNAs accumulates at the lowest level in the mannitol-treated *snrk2* decuple mutant seedlings (S6 Fig). There was no significant difference in SE protein abundance between the wild type and *snrk2* mutant (Fig 5B). To test whether the reduced HYL1 protein abundance might be due to a decreased *HYL1* transcript level, we performed qRT-PCR analysis with total RNA from rosette leaves of the *snrk2* triple mutant and wild type seedlings grown in soil but detected no obvious difference in the *HYL1 t*ranscript level between the two genotypes (Fig 5C). Similarly, *HYL1* transcript levels were not reduced in the *snrk2* decuple mutant seedlings grown in culture medium either before or after mannitol treatment (Fig 5D). Rather, the transcript levels were higher in the decuple mutant, especially after mannitol treatment (Fig 5D). These results strongly suggest that the SnRK2 kinases-mediated pathway modulates HYL1 protein abundance.

### The SnRK2 kinases may be associated with components of the miRNA processing complex

**Fig 6:**
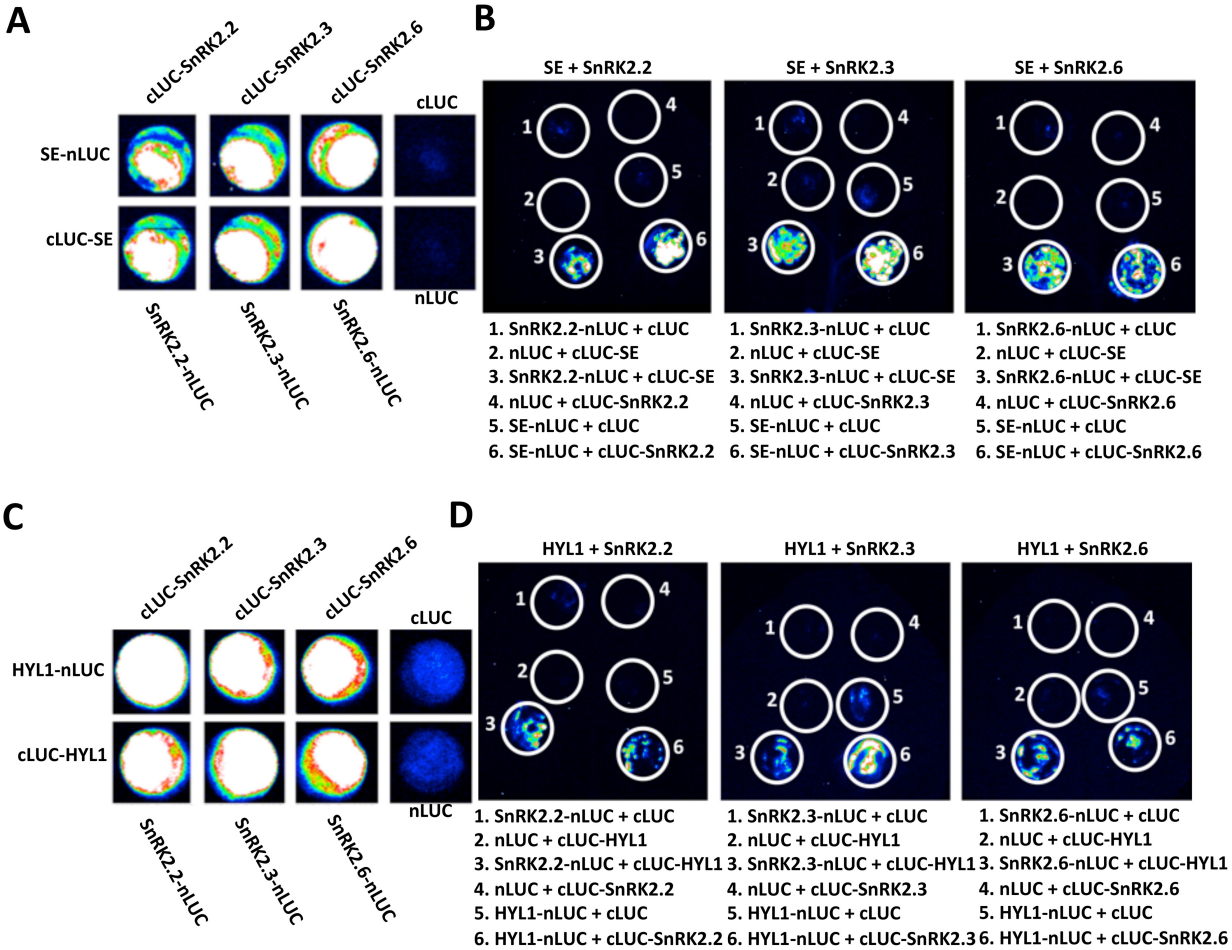
Members of SnRK2 family kinases may be associated with the miRNA biogenesis components SE and HYL1. (A) and (C) Split luciferase assay of the interaction between SE (A) or HYL1 (C) and SnRK2s in wild type *Arabidopsis* protoplasts. (B) and (D) Split luciferase assay of the interaction between SE (B) or HYL1 (D) and SnRK2s in infiltrated *N*. *benthamiana* leaves.

We speculated that a possible mechanism by which the SnRK2 kinases regulate miRNA biogenesis might involve a direct physical interaction between SnRK2s with one or more key components of the miRNA processing complex. To test this hypothesis, we performed a yeast two-hybrid assay and found that among the ten SnRK2s, SnRK2.2, SnRK2.3 and SnRK2.6 interacted strongly with SE but not with HYL1 (S7 Fig). To verify the SnRK2-SE interaction in plant cells, we performed a split-luciferase complementation assay using *Arabidopsis* protoplasts co-expressing fusion proteins with C-terminal or N-terminal half of a luciferase (cLUC or nLUC). Such an assay not only confirmed the SnRK2.2/2.3/2.6-SE interaction but also revealed that additional SnRKs could also interact with SE in the *Arabidopsis* protoplasts (Fig 6A and S8 Fig). The SnRK2-SE interaction could also be validated by a split luciferase assay in tobacco (*Nicotiana benthamiana*) leaves (Fig 6B). Interestingly, although our yeast two-hybrid assay failed to detect any SnRK2-HYL1 interaction, the split-luciferase assay in both *Arabidopsis* protoplasts and tobacco leaves revealed that HYL1 may also interact with the SE-interacting SnRK2s (Fig 6C and 6D and S8 Fig). Taken together, these results indicate that SnRK2 kinases may be associated with key components of the *Arabidopsis* miRNA processing complex.

### SE and HYL1 are potential substrates of SnRK2s

**Fig 7:**
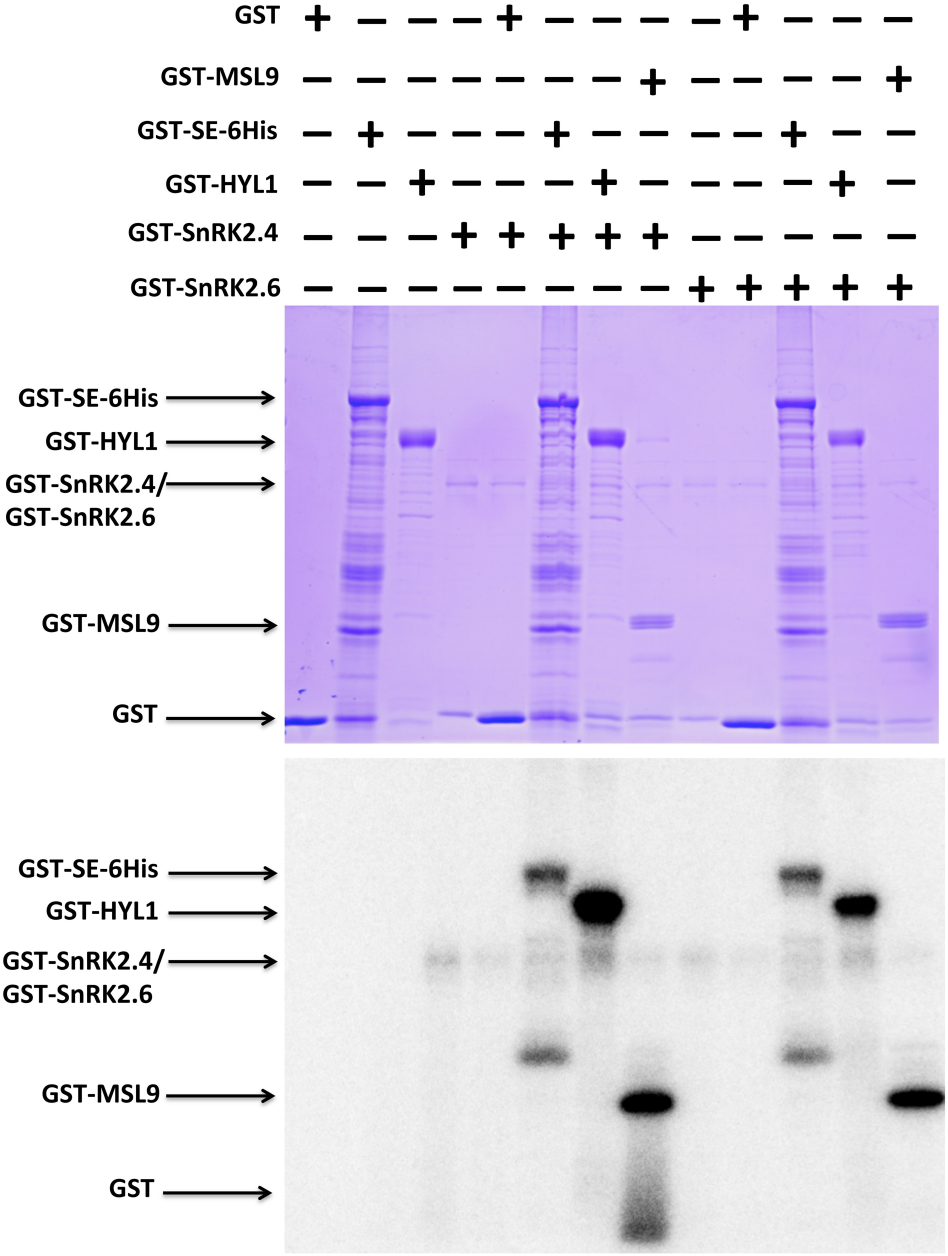
SE and HYL1 are substrates of SnRK2 kinases *in vitro*. *In vitro* phosphorylation assay of SE and HYL1 by SnRK2.4 and SnRK2.6 kinases. The phosphorylation levels were revealed by autoradiography (bottom panel) while the amounts of proteins used in the assay were visualized by Coomassie blue staining (top panel) of the SDS-PAGE gel. The GST and GST-MSL9 (Mechanosensitive channel of small conductance-like 9) were used as the negative and positive controls.

Our finding that SnRK2 kinases interact with SE in the yeast two hybrid assay and split luciferase assays coupled with earlier reports of SE being phosphorylated at multiple sites *in vivo* [40, 46–48] prompted us to examine if SE could be a direct SnRK2 substrate. Recombinant SE protein with an N-terminal GST tag and a C-terminal 6X His tag and recombinant SnRK2.4/2.6 kinases were produced and used in an *in vitro* phosphorylation assay. The assay showed that the two SnRK2 kinases, despite exhibiting weak *in vitro* autophosphorylation activities, were capable of strongly phosphorylating SE (Fig 7 and S9 Fig) and the positive control, MSL9, a recently discovered substrate of SnRK2.6 [47]. Because HYL1 could also bind with the SE-interacting SnRK2s in both *Arabidopsis* protoplasts and tobacco leaves (Fig 6C and 6D), we tested whether HYL1 may also be a SnRK2 substrate. A similar *in vitro* phosphorylation assay using recombinant HYL1 and SnRK2.4/2.6 proteins revealed that both SnRK2 kinases could strongly phosphorylate HYL1 (Fig 7 and S9 Fig). The K33R and K50N mutations in the catalytic domains of SnRK2.4 and SnRK2.6 respectively, abolished their phosphorylation of SE and HYL1 (S9 Fig). Therefore, SE and HYL1 are potential SnRK2 substrates.

## Discussion

In plants, miRNAs regulate the expression of genes involved in different biological processes. The physiological functions of these miRNAs have been extensively studied. However, how miRNA levels are modulated, especially how the key components of miRNA biogenesis are regulated remains largely unknown. miRNA biogenesis could be regulated at different steps. One of the most important regulatory steps of miRNA biogenesis is to control *MIR* gene transcription. The promoters of intergenic miRNAs have all the features associated with Pol II transcription and Pol II could be recruited to a *MIR* promoter to regulate the *MIR* transcription [49]. The transcription of intronic miRNA, which is encoded within an intron of a protein coding gene, could be regulated by the promoter of its host genes. Alternative splicing also acts as a regulatory mechanism in the modulation of some miRNAs. In *Arabidopsis*, miR400 is located in an intron and is co-transcribed with its host gene. Under heat stress conditions, alternative splicing results in the excision of a small fragment from the intron, and the remaining *MIR400* transcript-containing fragment is retained in the mRNA of its host gene, leading to higher abundance of *MIR400* transcript but lower expression of the mature miR400 [50]. Recent work has shown that cellular signaling pathways can also fine-tune miRNA biogenesis through posttranslational modifications of miRNA processing factors. In humans, mitogen-activated protein kinase (MAPK) Erk mediated phosphorylation of TRBP could enhance the DICER-TRBP complex stability and hence increase miRNA accumulation [51]. In *Arabidopsis*, the dephosphorylation of HYL1 by CPL1 is required for accurate miRNA processing and strand selection from miRNA duplexes [20]. A well-studied regulator of photomorphogenesis, constitutive photomorphogenic 1 (COP1), was found to affect miRNA accumulation through regulation of HYL1 [52]. In this study, we showed that SnRK2 kinases affect miRNA expression, possibly by modulating key components of the *Arabidopsis* miRNA processing complex.

SnRK2 regulation of miRNA expression may be executed by at least two different mechanisms. One is the SnRK2 mediated activation of ABA-responsive element binding factors (ABFs) and some other transcription factors, and the other is the SnRK2 mediated post-translational regulation of miRNA biogenesis pathway.

Regarding the former mechanism, the regulation is mainly at the transcriptional level. Once the ABFs and other transcription factors are activated by SnRK2s, they could directly regulate the transcription of *MIR* genes. Previous studies have shown that ABA could induce the accumulation of both mature miR168 and its precursor, and the ABF transcription factors could bind to the *MIR*168a promoter [53]. In addition to miR168, several other miRNAs were also found to be affected by ABA and several abiotic stresses and play important roles in the plant stress response. For example, disruption of miR159 mediated repression of *MYB33* and *MYB101* was found to alter ABA response during seed germination [54], while ABA-triggered induction of miR160 is very important for seed germination and early seedling development [55]. ABA could also reduce miR169 abundance, leading to elevated transcript level of *NUCLEAR FACTOR Y A5* (*NFYA5*), which positively regulates drought resistance [56]. Further investigation is needed to determine whether ABA regulation of these miRNAs is also mediated by SnRK2 activation of ABFs and other transcription factors. Some transcription factors, such as CDF2 [16], can modulate miRNA accumulation. It is unclear whether SnRK2s are involved in the phosphorylation of these factors and thereby affect the accumulation of miRNAs.

The second possible mechanism of the SnRK2-mediated regulation of miRNA expression likely involves posttranslational modifications of key components of the miRNA processing complex. Maintaining proper levels of miRNAs requires optimal levels of miRNA-processing components such as HYL1, whose protein abundance and/or full activities could be regulated by the SnRK2 kinases. We discovered that the *snrk2*.*2/3/6* triple mutation significantly reduces the protein abundance but not the transcript level, of HYL1 in the rosette leaves of soil-grown plants, indicating crucial roles of the three SnRK2s in regulating the HYL1 protein abundance. Our experiments also revealed the involvement of other SnRK2 kinases in controlling the HYL1 protein level in cultured whole seedlings, especially under osmotic stress known to activate nearly all 10 members of the SnRK2 family. It remains to be investigated whether the significant reduction of HYL1 abundance in the rosette leaves of soil-grown *snrk2*.*2/3/6* triple mutant or mannitol-treated seedlings of the *snrk2* decuple mutant is due to increased degradation or decreased protein synthesis of HYL1. Recently, Cho et al also found that the level of HYL1 is highly diminished in a *cop1* mutant background and suggested involvement of yet to be identified protease X in the proteolysis of HYL1 [52]. It would be interesting to know if there is a potential biochemical link between SnRK2-mediated HYL1 stabilization and COP1-mediated HYL1 stabilization. Given the fact that both HYL1 and SE may interact with and can be phosphorylated by the SnRK2 kinases *in vitro*, future studies should determine whether the SnRK2s phosphorylate HYL1 and SE *in vivo*, and if so, what kind of biochemical effect of the SnRK2-catalyzed phosphorylation is on the miRNA biogenesis factors and on miRNA accumulation. The SnRK2s mediated phosphorylation of SE might affect its localization or its interaction with other factors, rather than its protein abundance, since SE protein abundance was not affected substantially in the *snrk2* mutant. The phosphorylation could also regulate the biochemical activity of HYL1. A recent study showed that the phosphorylation status of HYL1 modulated by the phosphatase CPL1 is important for the normal function of HYL1 [20]. Seven serine residues were predicted as potential phosphorylation sites, and the phosphorylation status of at least two of them, serine-42 and serine-159, was shown to affect the recruitment of HYL1 to the processing complex [20]. A mass spectrometry experiment of purified HYL1 protein only identified the C-terminal repetitive peptide EAAFGSVETEK as being phosphorylated [20]. It is quite possible that HYL1 is a short-lived protein and very unstable [52], and only the six tandem repeats of this peptide could be detected. It will be very interesting to investigate if these phosphorylation sites are phosphorylated by the SnRK2 kinases in vivo. The HYL1 could also be phosphorylated by MPK3, which could not phosphorylate the serine-42 and serine-159 residues [29]. Detailed biochemical experiments are needed to map the in vivo phosphorylation sites of HYL1 and to determine their functional significance in regulating the protein stability and/or biochemical activities of HYL1. Our finding that key components of the miRNA processing complex are potential substrates of SnRK2 kinases suggests that the regulation of miRNA biogenesis is an important part of plant responses to ABA and osmotic stress. Consistent with this notion, the *hyl1-2* mutant was known to be hypersensitive to ABA [57].

The effect of SnRK2 kinases on miRNA accumulation might not only be related to its regulation of HYL1 protein abundance. HYL1 has dual roles in miRNA processing, i.e. miRNA processing efficiency and accuracy, and these functions can be uncoupled [58]. Our results show that as expected, miRNA processing accuracy was affected in *hyl1-2* (S4 Fig). However, miRNA processing accuracy was not affected substantially in the *snrk2*.*2/3/6* mutant (S4 Fig). This suggests that the reduced HYL1 protein abundance in *snrk2*.*2/3/6* mutant was sufficient to cause a decrease in miRNA processing efficiency but not in miRNA processing accuracy, i.e. an effect on miRNA processing accuracy may become obvious only when HYL1 protein abundance is further reduced. In addition, the effect of SnRK2-mediated regulation of other miRNA biogenesis factors such as SE should also be taken into consideration.

For both mechanisms, whether a SnRK2 kinase is activated or not determines the levels of some miRNAs. When grown in soil, the *snrk2*.*2/3/6* triple mutant grows poorly and the *snrk2* decuple mutant could not survive. However, both the *snrk2* triple and decuple mutants grow similarly to wild type in culture media under normal growth conditions [33, 35]. This is likely caused by activation of the SnRK2 kinases by the soil-growth condition but no or weak activation of SnRK2s by the medium-growth condition. Thus, for those miRNAs that are regulated by the SnRK2 kinase pathway, their abundance can be finely tuned by the degree of SnRK2 activation, which regulates the abundance and/or activities of transcriptional factors and the key components of the miRNA biogenesis machinery. Therefore, in response to various environmental stresses, the SnRK2 kinases are activated to reprogram gene expression not only by regulating transcription factors but also by modulating miRNA biogenesis.

## Materials and methods

### Plant materials and growth conditions

All *Arabidopsis* mutants and transgenic plants used in this study were in the Columbia-0 (Col-0) ecotype. Plants were grown in culture medium (Murashige-Skoog salts, 1% (w/v) sucrose and 0.6% (w/v) agar) with high humidity (with visible condensation in tissue culture plastic box), or in soil at 23°C under a 16h light/8h dark cycle, with a relative humidity ranging from 30% to 60%. For protoplast analysis, plants were grown under short photoperiod (12 hours of light at 23°C and 12 hours of dark at 20°C), low light (around 100 μE m^–2^ s^–1^), and relative humidity (50% to 70%) conditions. *snrk2*.*2/2*.*3*, *snrk2*.*6*, *snrk2*.*2/3/6*, *snrk2*.*1/4/5/7/8/9/10*, *snrk2*.*1/2/3/4/5/6/7/8/9/10*, *hyl1-2*, *se-1* have been described [33, 35, 59, 60].

### Plasmids construction

Detailed information on all plasmids and relevant primers is listed in S1 and S2 Tables.

### ABA and mannitol treatments

ABA or Mannitol treatment was performed as previously described [33, 35]. 50μM ABA or 800mM mannitol was added into the liquid culture and seedlings were removed at different time points.

### RNA analysis

Total RNAs were extracted using the RNeasy mini kit (Qiagen), and subsequently treated with DNase I to remove any genomic DNA contamination. Purified RNAs were used for reverse transcription with a High-Capacity cDNA Archive Kit (Applied Biosystems). SYBR Green PCR master mix kit was used for qRT-PCR analysis according to the manufacture’s instructions. Actin mRNA was used as an internal control. Relative gene expression level was calculated from the 2^-ΔΔCt^ values.

Mature miRNA quantification was performed according to TaqMan Small RNA Assays protocol (Applied Biosystems). The internal control was *Arabidopsis* SnoR101. qRT-PCR was performed using the TaqMan Gene Expression Master Mix (Applied Biosystems).

For small RNA gel blot analysis, total RNA was prepared by Trizol (Invitrogen), resolved by 15% PAGE under denaturing conditions (8M urea), electro–blotted and subsequently cross-linked onto Hybond-N^+^ (GE Healthcare) membrane. γ-^32^ATP-radiolabeled single-stranded DNA oligonucleotides complementary to the analyzed small RNAs were used as probes to detect specific miRNAs. See S2 Table for oligonucleotide primers and probes.

For small RNA deep sequencing, RNA samples of two biological replicates extracted from rosette leaves of 3-week-old plants grown in soil were used. Library preparation and deep sequencing with Illumina Hiseq 2500 were performed at Genomics Core Facility of Shanghai for Plant Stress Biology (Shanghai, China). Clean reads from raw small RNA sequences were generated by trimming adaptor sequences. Only the reads with sizes ranging from 18nt to 30nt were retained and mapped to the Arabidopsis genome (TAIR10). Reads identical to the annotated Arabidopsis mature miRNAs in the miRBase (release 21) were identified as mature miRNAs for subsequent analysis. Read counts of miRNAs [Reads Per Ten Million (RPTM) were calculated by normalizing miRNA counts with the total abundance of genome-matched small RNA reads in the corresponding small RNA library. Deep sequencing datasets were deposited into NCBI with accession No. GSE93243.

### Protein analysis

Proteins were extracted from seedlings or leaf samples with extraction buffer (50mM Tris-HCI [pH 7.5], 150mMNaCI, 1mM EDTA, 0.2% Triton X-100, 10% Glycerol, 1mM DTT, 1ug/ml Leupeptin, 1ug/ml Aprotinin, 1ug/ml Pepstatin A, 1mM PMSF, Phosphatase Inhibitor Cocktail Set II [Calbiochem]), separated by SDS-PAGE, and analyzed by immunobloting. Chemiluminescence signals were detected by autoradiography using ECL^™^ Prime Western Blotting System (GE Healthcare).

For the expression of recombinant proteins, all the recombinant proteins were expressed in *E*. *coli* BL21 strain overnight at 22°C induced by isopropyl β-d-thiogalactoside (IPTG). The His-fused proteins were purified with Ni^2+^ affinity column, whereas the GST-fused proteins were purified with glutathione sepharose resin (GE Healthcare). For *in vitro* phosphorylation assay, 0.2 μg of recombinant GST-tagged SnRK2.6 or GST-tagged SnRK2.4 and 1.0 μg substrate proteins were incubated in the presence of 50mM ATP and [λ-^32^P]ATP (0.1μCi per reaction) in 25 μl of reaction buffer (25mM Tris (pH7.5), 12mM MgCI_2_ and 1 mM DTT) at room temperature for 30 min. The reaction mixtures were separated by 10% SDS–polyacrylamide gel, visualized by Coomassie blue staining, and the phosphorylated proteins were analyzed by autoradiography.

For the yeast two-hybrid assay, Clontech strain AH109 was used. Genes of interest were cloned into pGBKT7 (Gal4 DNA binding domain, Clontech) or pGADT7 (Gal4 activation domain, Clontech). 3-amino-1,2,4-triazole (3-AT) was added to the selection medium to reduce the autoactivation of the reporter genes.

### Transient expression assay

For the split luciferase assay in *N*. *benthamiana*, genes of interest were cloned into vectors containing the C-terminal half of luciferase (cLUC) or the N-terminal half of luciferase (nLUC), transformed into *Agrobacterium tumefaciens* strain GV3101, and coinfiltrated into tobacco leaves. The infiltrated tobacco leaves were grown for an additional 2 or 3 days and used for analyzing the luminescent images. Methods for the similar assay using the Arabidopsis protoplasts were previously described [33].

## Supporting information

S1 Fig The *snrk2*.*2/3/6* mutant plants display leaf serration phenotype. (TIF)

S2 Fig Comparison of the expression of 24 nt siRNAs between wild type and *snrk2*.*2/3/6* mutant. (A) Relative abundance of 24 nt siRNAs in *snrk2*.*2/3/6* compared with wild type. Numbers of non-structural small RNAs are normalized based on total sequencing reads. (B) qRT-PCR analysis of the expression of 24 nt siRNAs in rosette leaves of wild type, *snrk2*.*2/3/6*, *hyl1-2* and *se-1*. qRT-PCR results are means ±SD of three biological replicates where the fold changes are normalized to the transcript level in WT.
(TIF)

S3 Fig Identical miRNAs with >2-fold abundance reduction (relative to the wild-type control)) in *snrk2*.*2/3/*6 mutants. (TIF)

S4 Fig Misprocessed reads at all highly expressed *MIRNA* loci. Imprecise miRNAs were defined as those that did not fall within ±2 bases of the annotated mature miRNA(s) or miRNA*(s) positions [58]. Black bars indicate medians.
(TIF)

S5 Fig Upregulated miRNA targets in ABA-treated *snrk2*.*2/3/6* seedlings. The expression level of miRNA targets in two-week-old wild type and *snrk2*.*2/3/6* seedlings treated with 10.0 μM ABA.
(TIF)

S6 Fig RNA blot hybridization of miRNAs from seedlings of wild type and *snrk2* mutants treated with mannitol. (TIF)

S7 Fig Yeast two-hybrid assay of the interaction between miRNA biogenesis components and members of SnRK2 kinase. –LT, medium without leucine and tryptophan; -LTH, medium without leucine, tryptophan and histidine. AD, GAL4 activation domain fusions; BD, GAL4 DNA binding domain fusions. Serial dilutions (10^−1^, 10^−2^, 10^−3^) of saturated cultures were spotted onto the plates. In the selection medium, 3-amino-1,2,4-triazole (3-AT) was added to reduce autoactivation.
(TIF)

S8 Fig The SnRK2 kinases may interact with miRNA processing factors. (A) Split luciferase assay of the interaction between SE and members of SnRK2 in wild type *Arabidopsis* protoplasts. (B) Split luciferase assay of the interaction between HYL1 and members of SnRK2 in wild type *Arabidopsis* protoplasts. (C) Split luciferase assay of the interaction between SnRK2.6 and SE or HYL1 in infiltrated *N*. *benthamiana* leaves (left) and wild type *Arabidopsis* protoplasts (right).
(TIF)

S9 Fig *In vitro* phosphorylation assay of SE and HYL1 by SnRK2.4 and SnRK2.6 kinases and their catalytic site mutants, SnRK2.4^K33R^ and SnRK2.6^K50N^. (TIF)

S1 Table Plasmids used in this study. (XLSX)

S2 Table Primers and oligonucleotides usd in this study. (XLSX)
