# Supplementary figures and images for: The SnRK2 kinases modulate miRNA accumulation in Arabidopsis
Source: PLoS Genet. 2017 Apr 18;13(4):e1006753. doi: 10.1371/journal.pgen.1006753 (PMC5413060; doi:10.1371/journal.pgen.1006753)

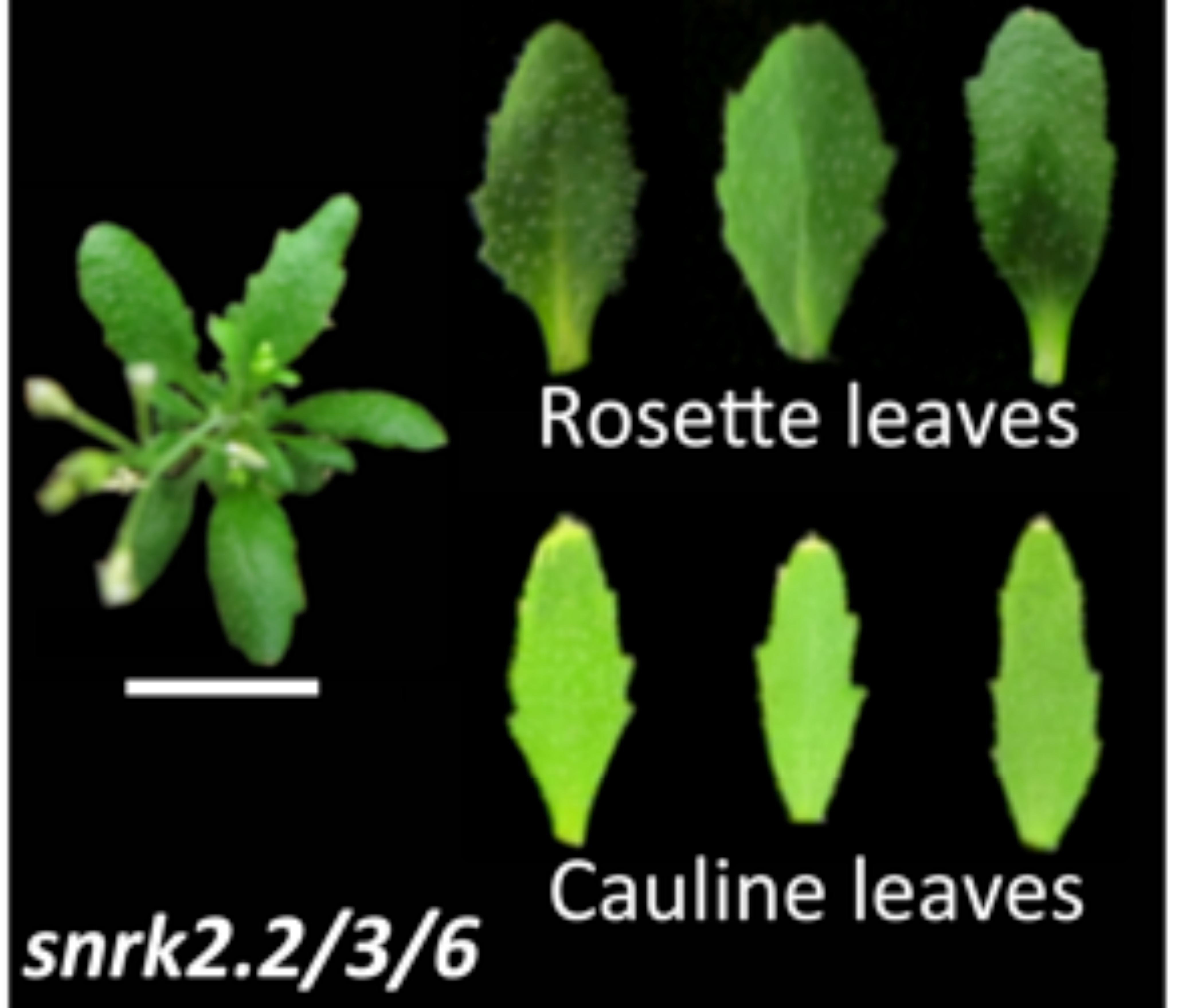

Supplement: S1 Fig — (TIF) [file pgen.1006753.s001.tif]

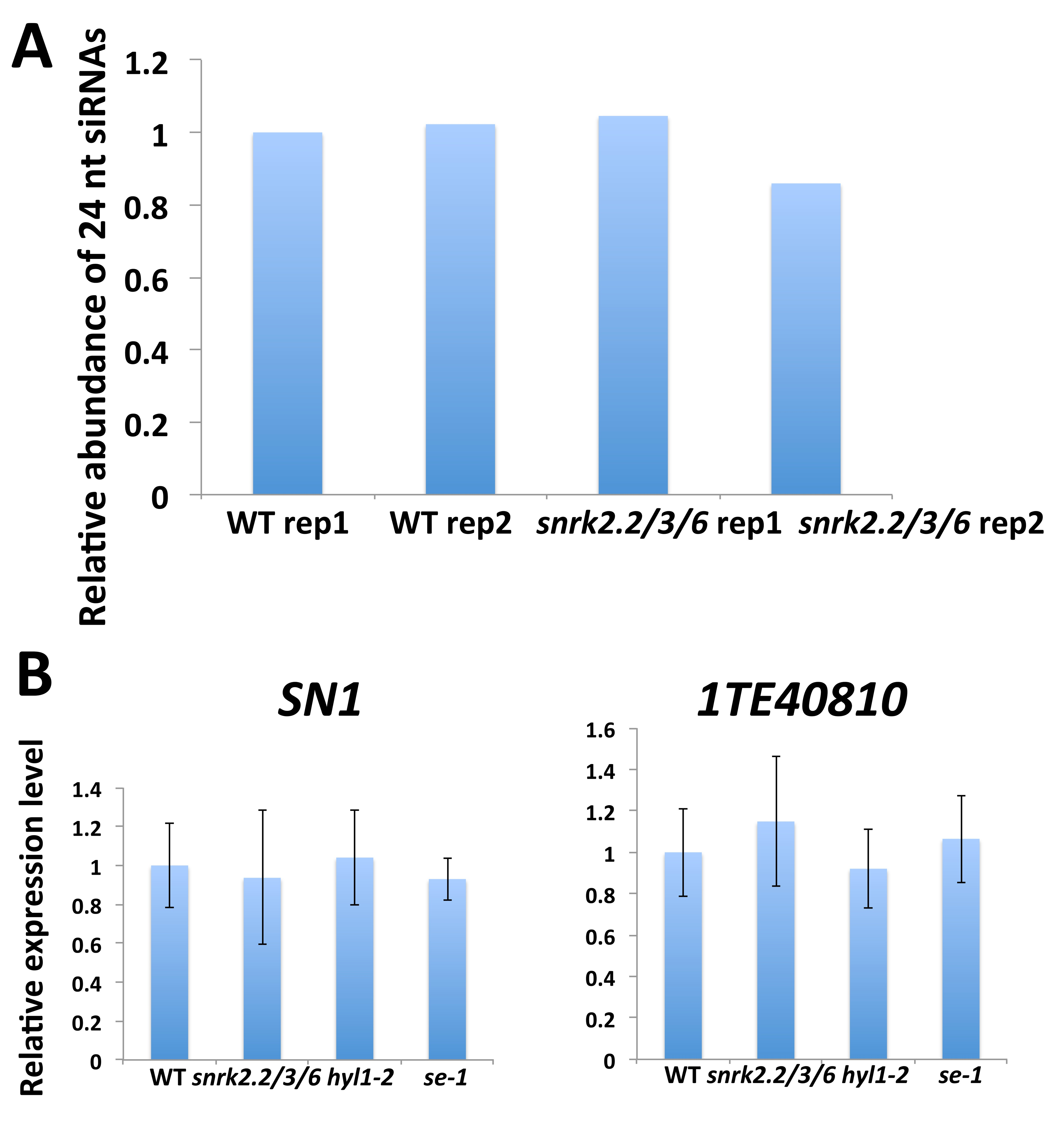

Supplement: S2 Fig — (A) Relative abundance of 24 nt siRNAs in snrk2.2/3/6 compared with wild type. Numbers of non-structural small RNAs are normalized based on total sequencing reads. (B) qRT-PCR analysis of the expression of 24 nt siRNAs in rosette leaves of wild type, snrk2.2/3/6, hyl1-2 and se-1. qRT-PCR results are means ±SD of three biological replicates where the fold changes are normalized to the transcript level in WT. (TIF) [file pgen.1006753.s002.tif]

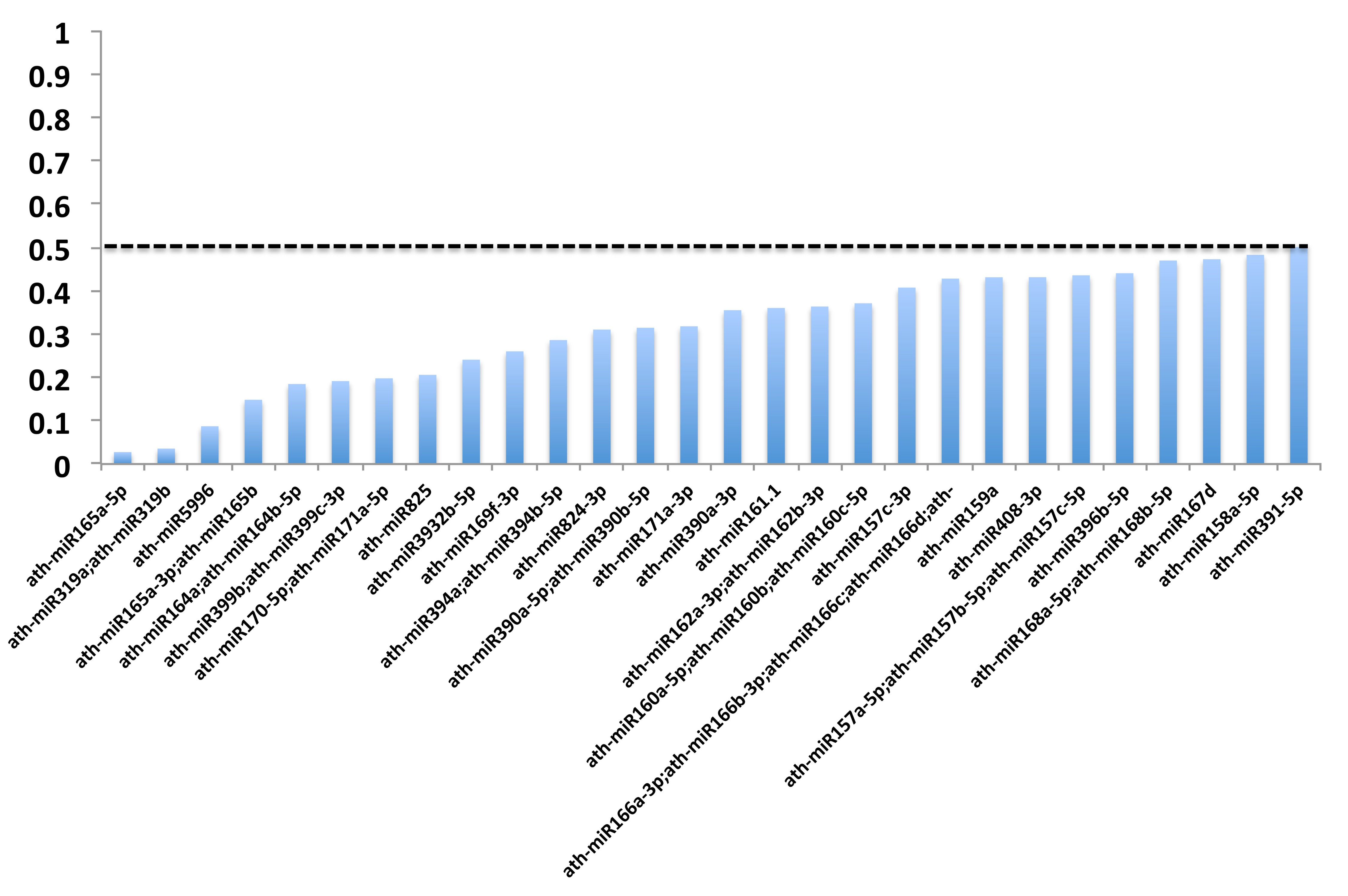

Supplement: S3 Fig — (TIF) [file pgen.1006753.s003.tif]

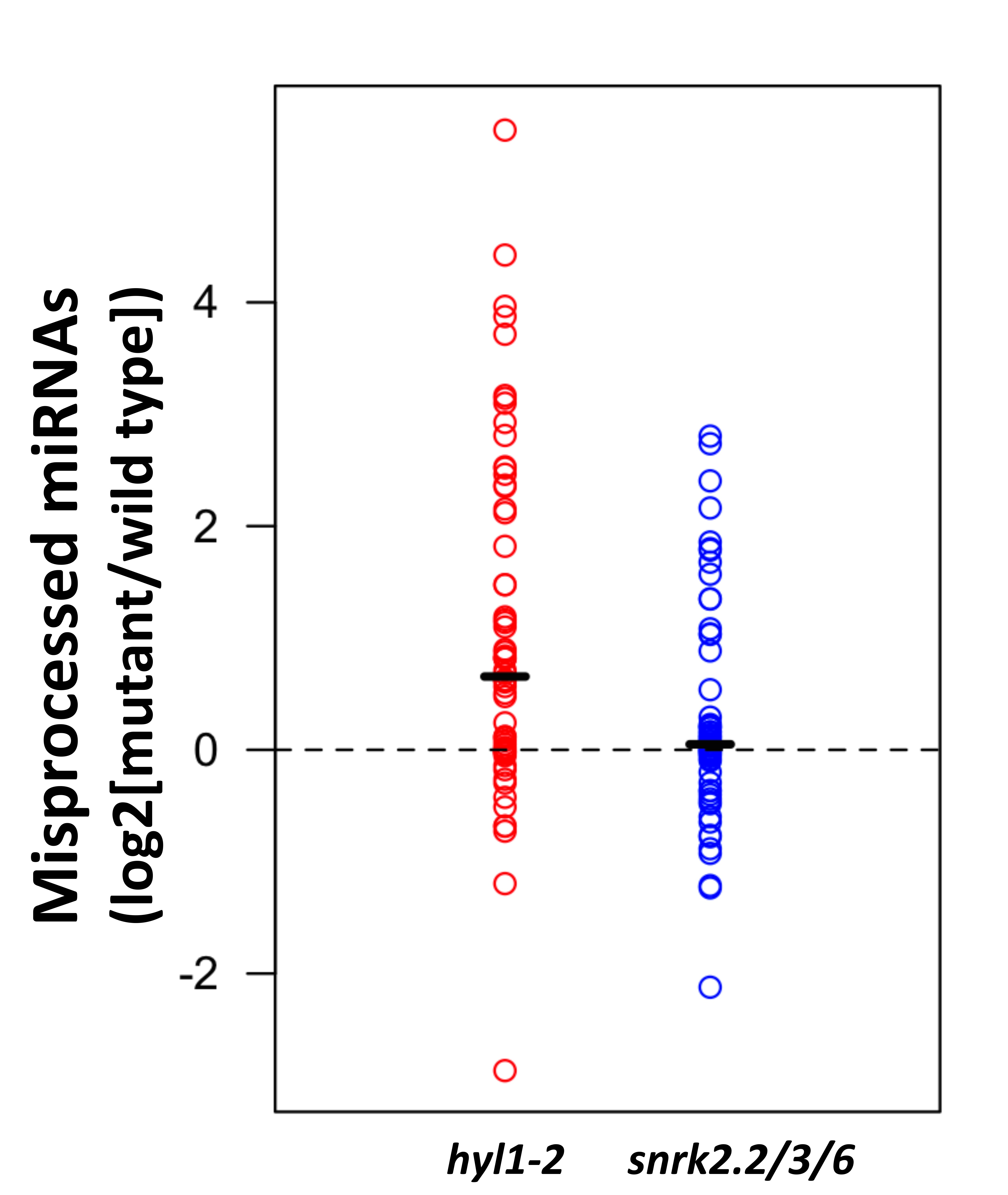

Supplement: S4 Fig — Imprecise miRNAs were defined as those that did not fall within ±2 bases of the annotated mature miRNA(s) or miRNA*(s) positions [58]. Black bars indicate medians. (TIF) [file pgen.1006753.s004.tif]

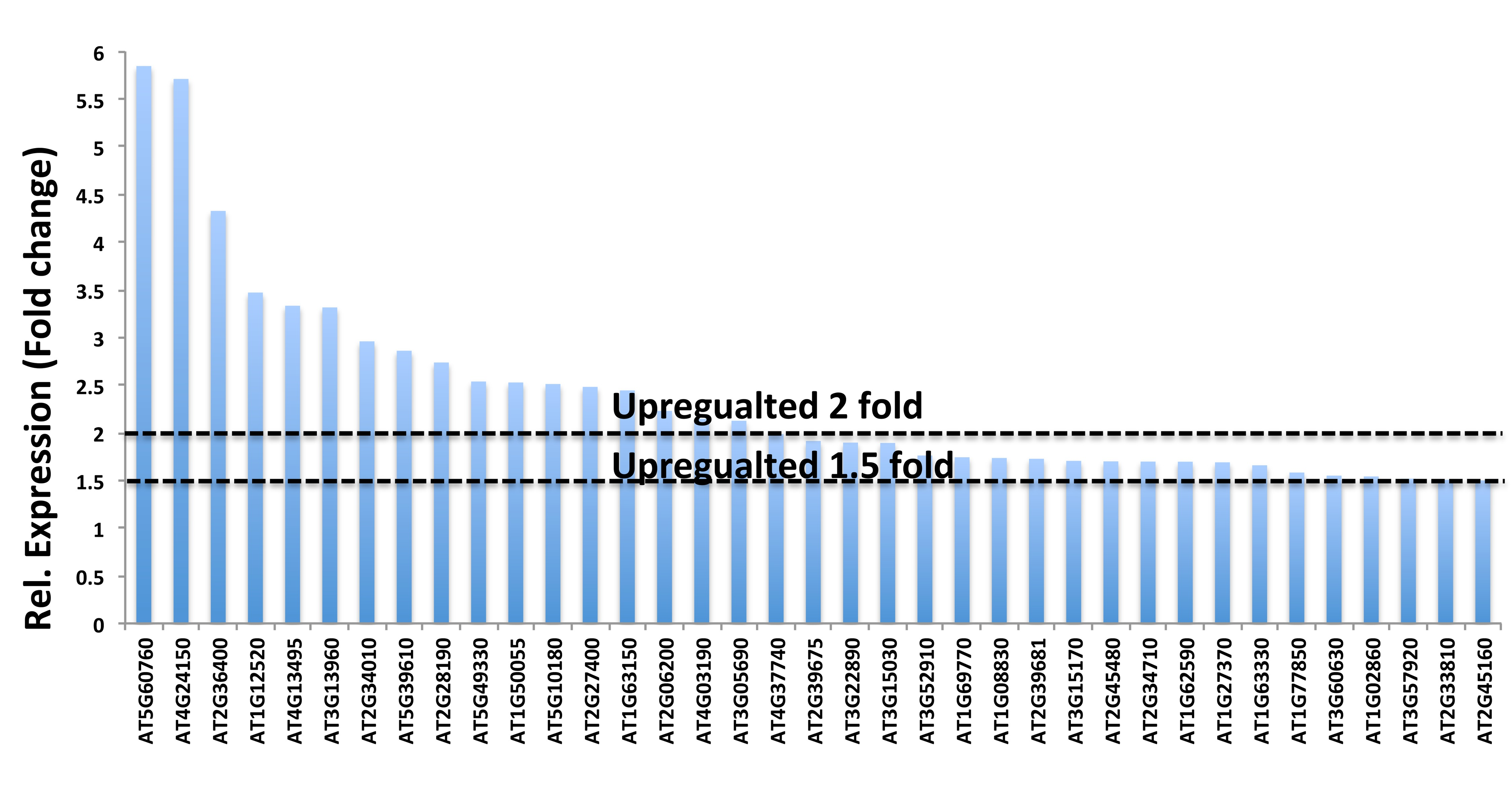

Supplement: S5 Fig — The expression level of miRNA targets in two-week-old wild type and snrk2.2/3/6 seedlings treated with 10.0 μM ABA. (TIF) [file pgen.1006753.s005.tif]

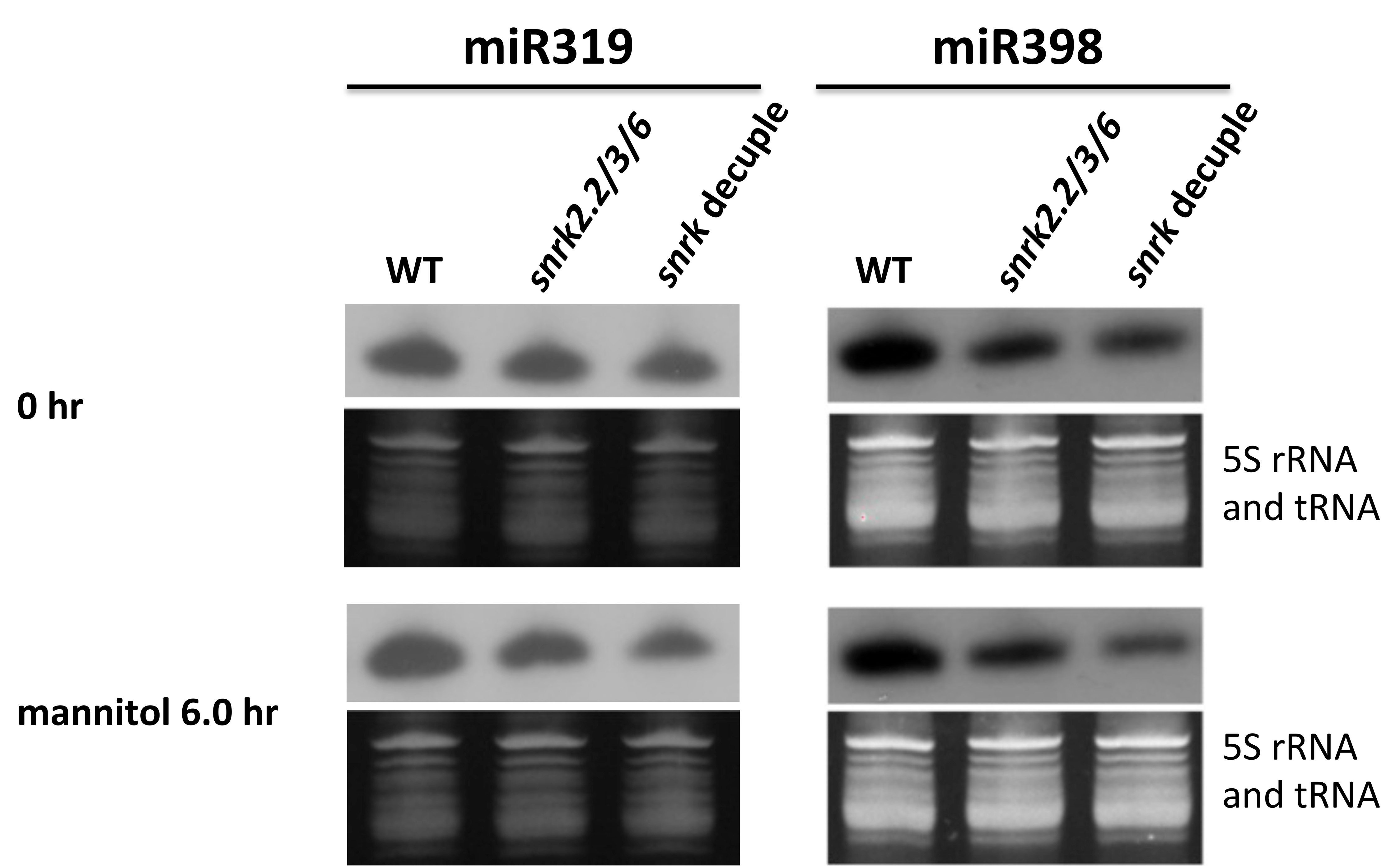

Supplement: S6 Fig — (TIF) [file pgen.1006753.s006.tif]

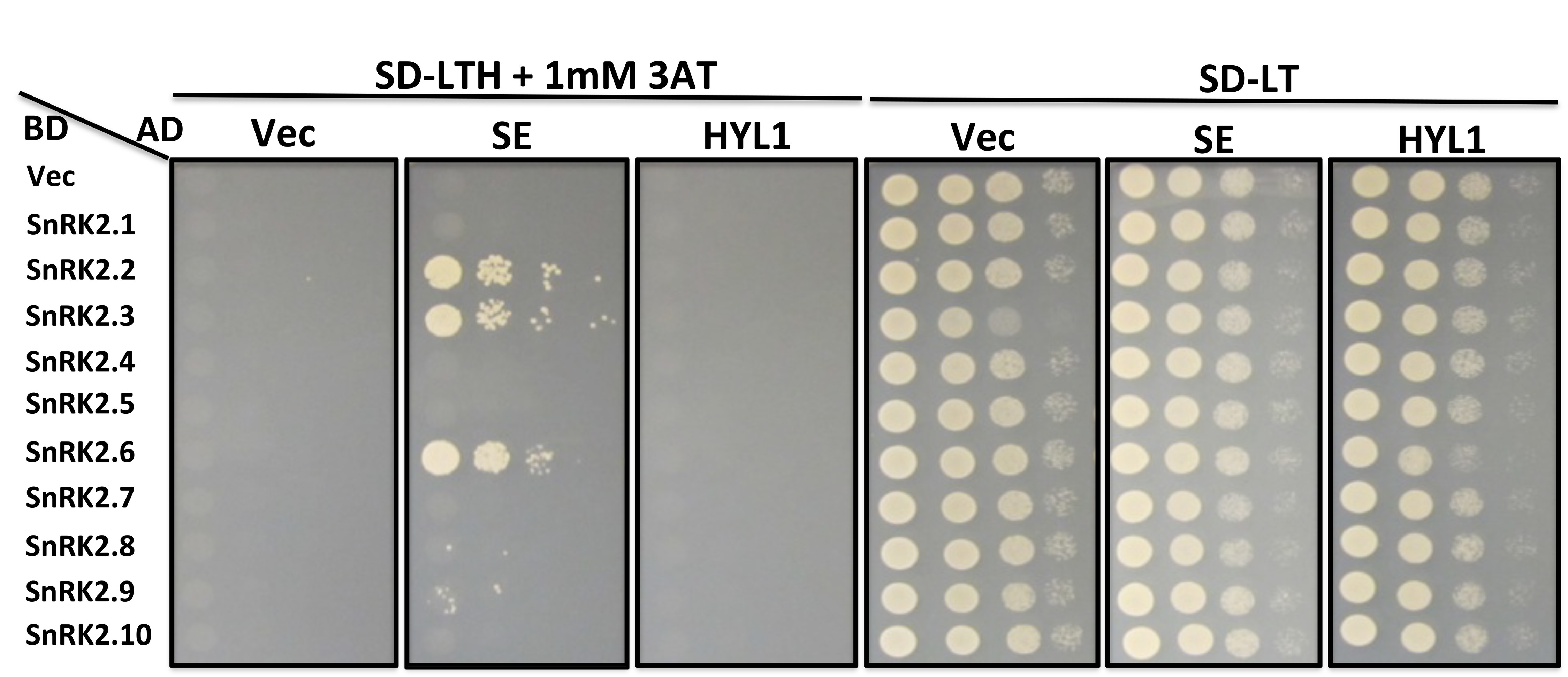

Supplement: S7 Fig — –LT, medium without leucine and tryptophan; -LTH, medium without leucine, tryptophan and histidine. AD, GAL4 activation domain fusions; BD, GAL4 DNA binding domain fusions. Serial dilutions (10−1, 10−2, 10−3) of saturated cultures were spotted onto the plates. In the selection medium, 3-amino-1,2,4-triazole (3-AT) was added to reduce autoactivation. (TIF) [file pgen.1006753.s007.tif]

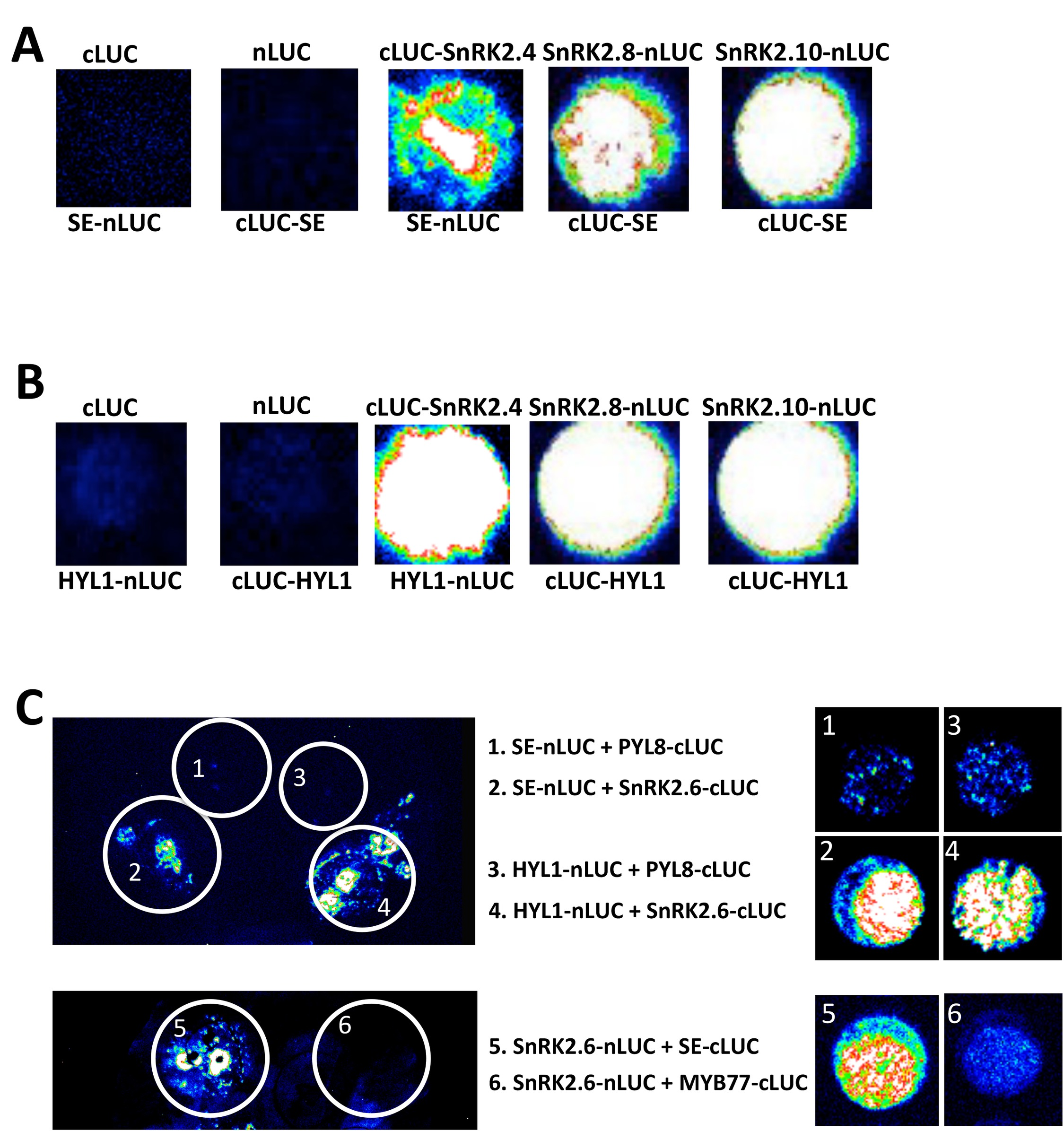

Supplement: S8 Fig — (A) Split luciferase assay of the interaction between SE and members of SnRK2 in wild type Arabidopsis protoplasts. (B) Split luciferase assay of the interaction between HYL1 and members of SnRK2 in wild type Arabidopsis protoplasts. (C) Split luciferase assay of the interaction between SnRK2.6 and SE or HYL1 in infiltrated N. benthamiana leaves (left) and wild type Arabidopsis protoplasts (right). (TIF) [file pgen.1006753.s008.tif]

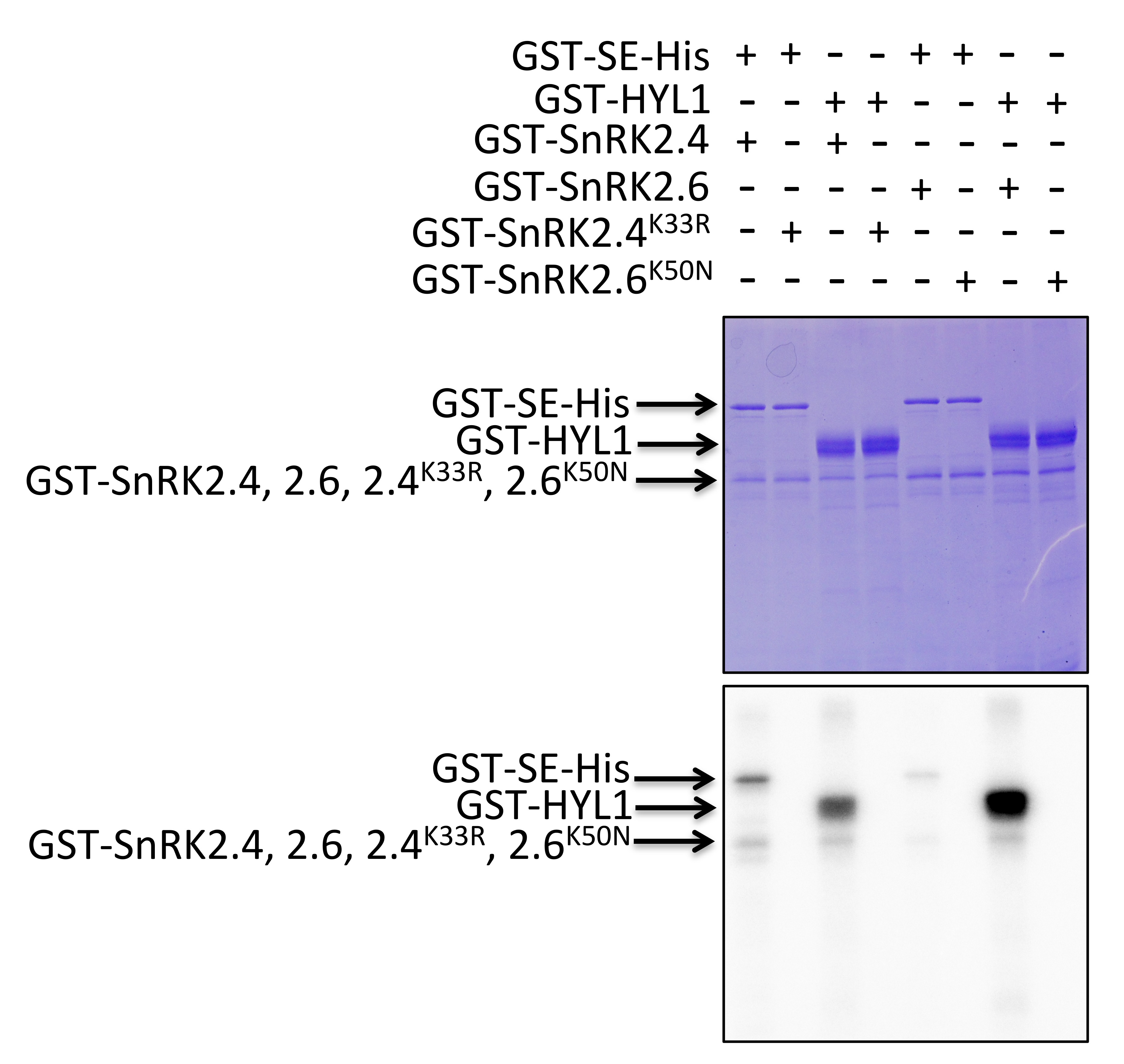

Supplement: S9 Fig — (TIF) [file pgen.1006753.s009.tif]
